# Influence of platelet-activating factor receptor (PAFR) on *Brucella abortus* infection: implications for manipulating the phagocytic strategy of *B. abortus*

**DOI:** 10.1186/s12866-016-0685-8

**Published:** 2016-04-21

**Authors:** Jin Ju Lee, Hannah Leah Simborio, Alisha Wehdnesday Bernardo Reyes, Huynh Tan Hop, Lauren Togonon Arayan, Hu Jang Lee, Wongi Min, Moon Her, Man Hee Rhee, Masahisa Watarai, Hong Hee Chang, Suk Kim

**Affiliations:** Animal and Plant Quarantine Agency, Anyang, Gyeonggi-do 430-757 Republic of Korea; Institute of Animal Medicine, College of Veterinary Medicine, Gyeongsang National University, Jinju, 660-701 Republic of Korea; College of Veterinary Medicine, Kyungpook National University, Daegu, 702-701 Republic of Korea; Department of Veterinary Public Health, Faculty of Agriculture, Yamaguchi University, Yamaguchi, 753-8515 Japan; Institute of Agriculture and Life Science, Gyeongsang National University, Jinju, 660-701 Republic of Korea

**Keywords:** *B. abortus*, Platelet-activating factor receptor, JAK2, Phagocytosis

## Abstract

**Background:**

*Brucella abortus* is an intracellular pathogen which can infect and persist in host cells through multiple interactions. Above all, its interaction to host cell receptor is important to understand the pathogenic mechanisms of *B. abortus*. Accordingly, we demonstrated that platelet-activating factor receptor (PAFR) affects host cell response against *B. abortus* infection.

**Results:**

First of all, *B. abortus* infection to macrophage induces secretion of platelet-activating factor (PAF), which is a PAFR agonist. The stimulation of PAFR by PAF remarkably increases *B. abortus* uptake into macrophages. It induces Janus kinase 2 (JAK2) and p38α phosphorylation, indicating that PAFR-mediated activation of JAK2 signaling leads to enhanced uptake of *B. abortus*. Moreover, the dynamics of F-actin polymerization revealed that PAFR-mediated *B. abortus* uptake is related with the reorganization of F-actin and JAK2. Upon *B. abortus* phagocytosis, reduced PAFR in the membrane and subsequently increased levels of PAFR colocalization with endosomes were observed which indicate that *B. abortus* uptake into macrophages allowed PAFR trafficking to endosomes.

**Conclusions:**

This study demonstrated that PAFR has a compelling involvement in *B. abortus* uptake as a promoter of phagocytosis, which is associated with JAK2 activation. Thus, our findings establish a novel insight into a receptor-related phagocytic mechanism of *B. abortus*.

**Electronic supplementary material:**

The online version of this article (doi:10.1186/s12866-016-0685-8) contains supplementary material, which is available to authorized users.

## Background

*Brucella* spp. are intracellular pathogens responsible for a chronic mammalian zoonotic disease, which has emerged as an ongoing public health problem worldwide [1]. These organisms cause subtle infections, which entail extensive replication inside host cells, such as macrophages, dendritic cells, and placental trophoblasts, for several days without producing toxic effects [2]. The virulence factors used by the *Brucella* spp. to invade and persist are assumed to aid the organism's ability to avoid the killing mechanisms within cells [3], but their molecular mechanisms are not fully understood. In macrophages, *Brucella* may bind to distinct phagocytic receptors, such as Fc gamma receptors and scavenger receptors, but may also bind to unknown receptors resulting in subsequent engulfment by zipper-like phagocytosis [4]. It has also been shown that *Brucella* invades phagocytes through lipid raft microdomains [5, 6].

Platelet-activating factor receptor (PAFR) is a member of the G protein-coupled receptor superfamily (GPCR) that is expressed on various cell types, including neutrophils, macrophages, monocytes, and epithelial cells [7]. This receptor is activated by its ligand, PAF, which is an effective phospholipid mediator with multiple physiological and pathological involvement in allergic disorders and inflammation [8, 9]. Both PAFR and PAF may enhance the host ability to manage infections by promoting phagocytosis and eradication of engulfed microorganisms. PAFR is engaged during the host response to overcome infections through promoting phagocytosis and the subsequent elimination of internalized pathogens [10, 11].

Janus kinase (JAK) 2 is generally known as a key linker of cytokine-mediated signal transduction and modulation of immune responses [12]. It has been elucidated that JAK2 is involved in the activation of Src-kinase, PI3K, MAPKs, and STAT downstream signaling following cytokine receptor activation and infection [13, 14]. JAK2 signaling is not a distinctive hallmark of cytokine receptors; however, some evidences support its significance in GPCR signaling with some studies showing that JAK2 is associated with angiotensin AT1 receptor [15] and PAFR [16].

It has been demonstrated that PAFR stimulation activates divergent signaling pathways. Whether *B. abortus* interacts with PAFR and what phagocytic mechanisms of *B. abortus* are associated with PAFR-linked signaling have not been defined. Here, we address a novel phagocytic mechanism and reveal that PAFR facilitates the phagocytosis of *B. abortus*, and this event is associated with intracellular JAK2 pathways leading to amplified rearrangement of the actin cytoskeleton. In correlation with the interaction of *B. abortus* with PAFR-linked JAK2 signaling, there is evidence that PAF production is notably increased by *B. abortus* infection, which triggers trafficking of PAFR from the membrane to the endosomes. Taken together, this study suggests that the PAFR-linked JAK2 signaling contributes to the entry of *B. abortus* into macrophages. These findings highlight an important receptor-mediated signaling pathway involved in the *B. abortus* phagocytic mechanism.

## Results

### Uptake of *B. abortus* contributes to PAF production by enhancing activation of LPCAT2 through JAK2-mediated pathway

**Fig. 1:**
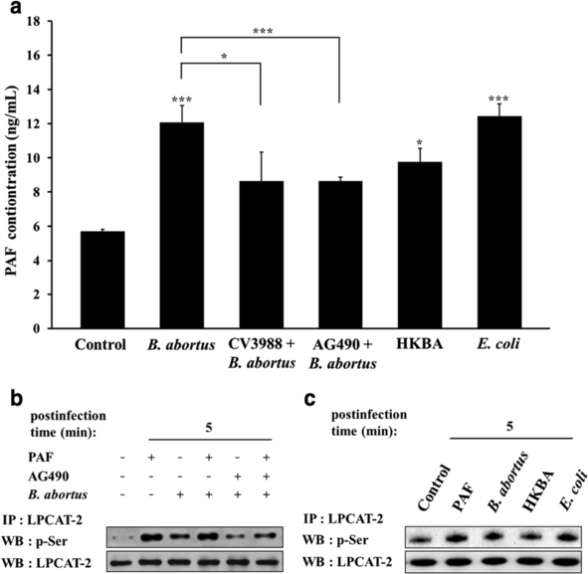
Production of PAF linked to LPCAT2 activation is enhanced by *B. abortus* infection. **(a)** RAW264.7 cells pretreated with AG490 (75 μM) or CV3988 (1 μM), a PAFR antagonist, for 1 h were infected with live *B. abortus* for 5 min. The cells were infected with heat-killed *B. abortus* and live *E. coli* O157:H7 for 5 min. The levels of PAF secretion in the culture supernatants were quantified by PAF ELISA. Data represent the mean ± SD of triplicate trials from three independent experiments. Statistically significant differences from the untreated samples are indicated by asterisks (*, *P* < 0.05; ***, *P* < 0.001). **(b)** Cells pretreated with PAF (200 nM), a PAFR agonist, for 5 min and AG490 (75 μM), a JAK2 inhibitor, for 1 h were infected with *B. abortus* for 5 min. **(c)** Cells were also were infected with heat-killed *B. abortus* or live *E. coli* O157:H7 for 5 min. The cells were processed for immunoprecipitation with a LPCAT2 antibody and then were probed with a phosphoserine antibody. The membrane was then stripped and re-probed with LPCAT2 antibody. The images shown are representative of three independent experiments

Previous studies elucidated the action of PAFR on bacterial infections [10, 17], however, an interaction between *B. abortus* and PAFR or its ligand, PAF, has not yet been investigated. We first determined whether *B. abortus* infection induces PAF production through activation of PAFR. The PAF content increased at a high level at 5 min post-infection with *B. abortus* (2.12-fold increase) compared to resting cells (Fig. 1a). PAF has been reported to stimulate PAFR which is associated with JAK2 signaling [18], and in our previous study, we demonstrated that JAK2 activation is involved in the invasion pathway of *B. abortus* [19]. Consequently, for comprehensive correlation between PAF production and *B. abortus* infection on PAFR-mediated signaling pathway, we measured PAF content in both CV3988 (PAFR antagonist)-treated and AG490 (JAK2 inhibitor)-treated cells during *B. abortus* uptake. As a result, the levels of PAF secretion during *B. abortus* infection were significantly attenuated in both CV3988- and AG490-treated cells, demonstrating approximately 1.40-fold reductions. Collectively, PAF production is enhanced during *B. abortus* infection through a JAK2-mediated pathway. Additionally, the PAF content was not significantly increased in cells infected with heat-killed *B. abortus* compared to resting cells or live *B. abortus*, while in the case of *E. coli* O157:H7 infection, it was remarkably increased (2.18-fold increase) as much as those of live *B. abortus*.

Phosphorylation of lyso-phosphatidylcholine acyltransferase 2 (LPCAT2), an enzyme involved in the synthesis of PAF from lyso-PAF, enhances PAF production in endotoxin-stimulated macrophages [20]. We hypothesized that *B. abortus* could augment the activation of LPCAT2 as a key step for PAF production, hence we investigated the phosphorylation of immunoprecipitated LPCAT2 in *B. abortus*-infected cells. As a result, *B. abortus* infection and PAF stimulation resulted in notable increase in the values of LPCAT2 activation by 53.63 % at 5 min post-infection compared to resting cells (Fig. 1b). In contrast, the LPCAT2 activation values were reduced to 35.65 % at 5 min post-infection in JAK2-inhibited cells. Moreover, the LPCAT2 activation was not significantly high in cells infected with heat-killed *B. abortus* compared to resting cells or live *B. abortus*, but that of *E. coli* O157:H7 infection showed an increase activation (1.37-fold) as much as that of live *B. abortus* (Fig. 1c). In correlation with the LPCAT2 phosphorylation data, these findings demonstrated that LPCAT2 is activated during uptake of *B. abortus* into macrophages mediated by JAK2 signaling pathway.

### PAFR-linked event triggers uptake of *B. abortus* into macrophages

**Fig. 2:**
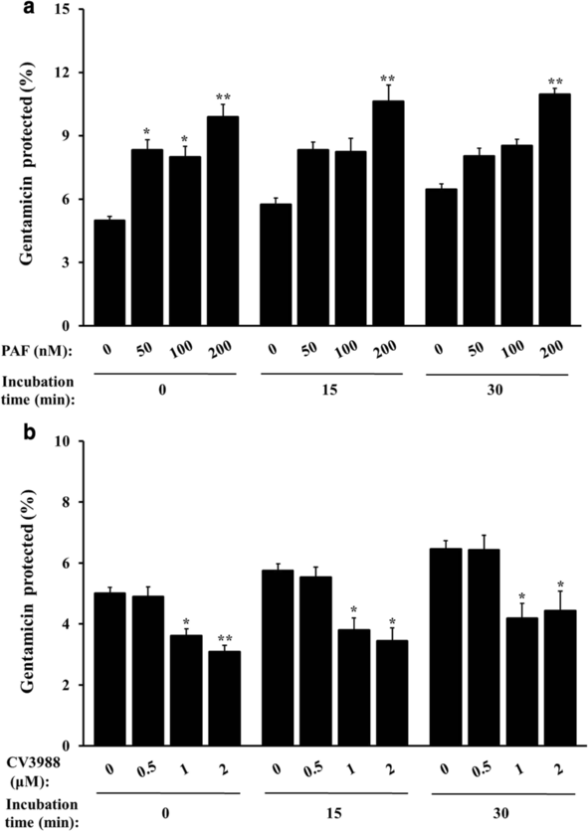
The stimulation of PAFR positively affects uptake of *B. abortus* into macrophages. **(a)** RAW 264.7 cells were pretreated for 5 min with serial concentrations (0–200 nM) of PAF, a PAFR agonist, followed by infection with *B. abortus* for the indicated times. **(b)** Cells were pretreated for 1 h with a serial concentration (0–2 μM) of CV3988, a PAFR antagonist, and then infected as described in (**a**). Bacterial internalization efficiency was determined by evaluating the protection of internalized bacteria from gentamicin treatment. All data represent the mean ± SD of triplicate trials from three independent experiments. Statistically significant differences from the untreated samples are indicated by asterisks (*, *P* < 0.05; **, *P* < 0.01; ***, *P* < 0.001)

Several studies indicated that *B. abortus*-induced PAF production is involved in PAFR-mediated signaling pathway, and accumulating studies have shown that PAFR has important roles for bacterial infections [10, 17]. Thus, we further investigated how PAFR is involved in the uptake of *B. abortus*. Macrophage cells were stimulated with various concentrations of PAF, which effectively acts through PAFR, in an artificial manner for a range of incubation times followed by infection with *B. abortus*. The uptake of *B. abortus* was significantly increased by the stimulation of PAF for 5 min in a dose-dependent pattern compared with untreated cells (*P* < 0.001) (Fig. 2a and Additional file 1: Table S1a). Conversely, the inhibition of PAFR function by the PAFR antagonist CV3988 resulted in notably reduced uptake of *B. abortus* in a dose-dependent pattern (*P* < 0.05) (Fig. 2b and Additional file 1: Table S1a). Additionally, when we tested the effects of PAFR activation on *B. abortus* internalization with different infection doses (MOIs of 10 and 100), the numbers of internalized bacteria were significantly increased at MOI 100 than MOI 10 in control cells but also in PAF-treated cells (Additional file 1: Table S1b). These results verify that PAFR activation by PAF contributes to the uptake of *B. abortus* into macrophages.

### PAFR-induced activation of JAK2 signaling is augmented by uptake of *B. abortus* into macrophages

**Fig. 3:**
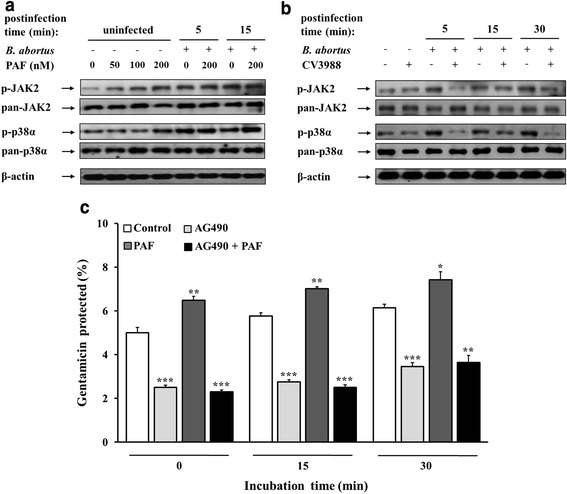
Activation of PAFR-associated JAK2 signaling involved in *B. abortus* uptake. **(a)** RAW 264.7 cells were pretreated for 5 min with a serial concentration (0–200 nM) of PAF. Of the PAF-treated cells, cells in 0 and 200 nM PAF were infected with *B. abortus* for 5 min. The cells were lysed, and the activation of JAK2 and p38α were monitored by immunoblot analysis. Images shown are representatives of three independent experiments. **(b)** Cells were pretreated with CV3988 (1 μM) for 1 h, followed by infection with *B. abortus* for the indicated times (5, 15, or 30 min), and then monitored by immunoblot analysis as described in (**a**). **(c)** Cells pretreated with or without AG490 (75 μM) for 1 h were stimulated with PAF (200 nM), and then the infection assay was conducted using the same procedure used to evaluate bacterial uptake into macrophages. Statistically significant differences from the untreated samples are indicated by asterisks (**, *P* < 0.01; ***, *P* < 0.001)

Some studies have found that stimulation of PAFR by PAF activates JAK2 as demonstrated in a mutant PAFR which failed to activate JAK2 phosphorylation [12, 16]. Previously, we confirmed that the activation of JAK2 and downstream proteins were induced during the uptake of *B. abortus* into macrophages [19]. Thus, we investigated the phosphorylation of JAK2 and the downstream protein p38α to verify whether PAFR-induced JAK2 activation is associated with *B. abortus* uptake by macrophages. In agreement with previous findings, JAK2 phosphorylation was increased upon *B. abortus* infection, and the stimulation of PAFR by PAF boosts JAK2 activation (increased values of 14.78 % at 5 min and 14.49 % at 15 min) during *B. abortus* uptake (Fig. 3a). In contrast, the suppression of PAFR by CV3988 resulted in no considerable difference in the phosphorylation state of JAK2 compared with the resting state of PAFR, but JAK2 activation was hindered (reduced values of 40.51 % at 5 min, 27.04 % at 15 min, and 24.20 % at 30 min) upon *B. abortus* infection (Fig. 3b). These findings indicate that stimulation of PAFR upon infection of *B. abortus* in macrophages induces activation of JAK2.

### Interaction of PAFR with intracellular JAK2 signaling assisted uptake of *B. abortus* into macrophages

Together with the supporting data referring to PAFR-related JAK2 signaling [18], we investigated whether *B. abortus* entry is involved in the interaction of PAFR with intracellular JAK2 signaling. Consequently, we examined whether the attenuation of *B. abortus* uptake by inhibition of JAK2 is altered by stimulation or interference of PAFR. The progressively increased uptake of *B. abortus* by PAF stimulation in untreated cells (*P* < 0.001), in contrast to the reduced uptake in JAK2-inactivated cells, was slightly altered by PAF stimulation (Fig. 3c and Additional file 1: Table S1a). These results suggested that PAFR is linked to intracellular JAK2, which acts as its downstream signal, and has an important role in the uptake process of *B. abortus* into macrophages. Collectively, these findings suggest that PAFR interacts with intracellular JAK2 signaling for the uptake of *B. abortus* into macrophages.

### PAFR has a positive effect on phagocytosis of *B. abortus* into macrophages and amplifies actin polymerization

**Fig. 4:**
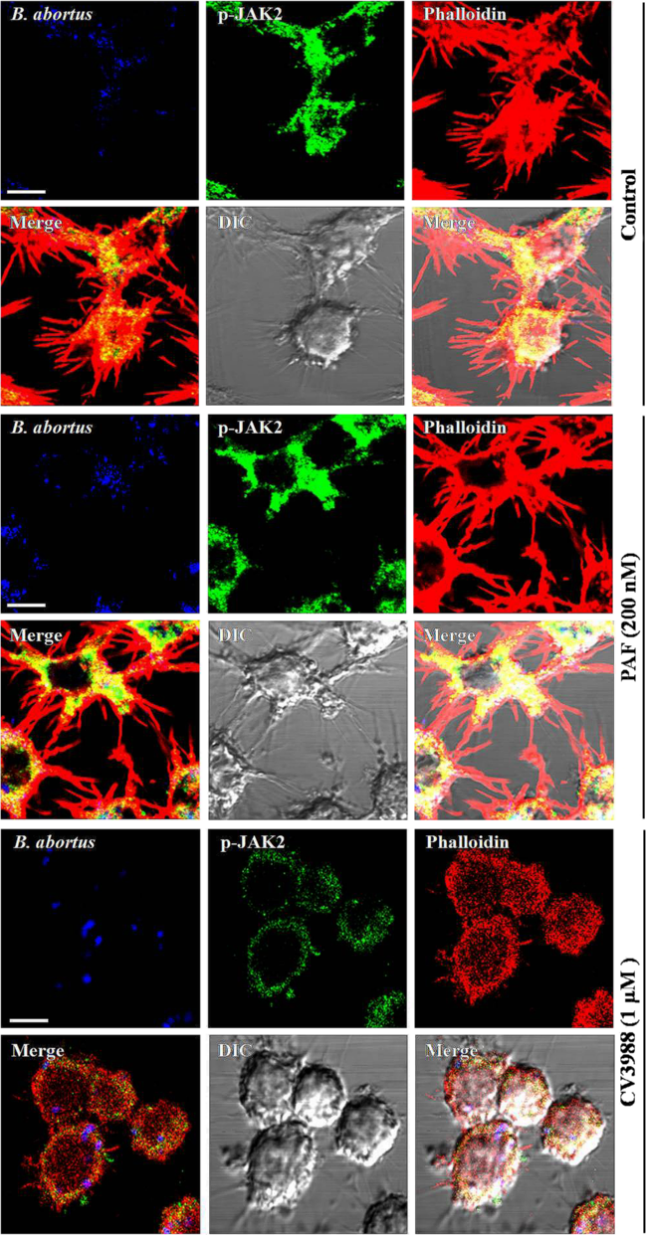
PAFR cooperates with JAK2 signaling for phagocytosis of *B. abortus*. RAW 264.7 cells were pretreated with or without PAF (200 nM) for 5 min or CV3988 (1 μM) for 1 h prior to infection; the cells were then infected with Alexa Fluor 405-labeled *B. abortus* (blue) for the indicated times. To observe redistribution of F-actin polymerization and activated JAK2, the cells were fixed and stained with both rhodamine-conjugated phalloidin for F-actin (red) and FITC-labeled phospho-JAK2 antibody (green) immediately following a 10 min infection. The merged images are also shown and all scale bars in images represent 10 μm. All results are representatives of three separate experiments

The association of PAFR with phagocytosis illuminated the function of PAFR as an infection control protein [11, 21], and the recruitment of PAFR with intracellular kinases elicits distinct actin rearrangement [22, 23]. Furthermore, we previously demonstrated that *B. abortus* uptake was accompanied by activation of JAK2 occurred through F-actin polymerization [19]. Thus, to determine whether JAK2 activation-associated actin polymerization in the phagocytosis of *B. abortus* is affected by PAFR function, we first visualized F-actin polymerization during *B. abortus* invasion into PAFR-stimulated or PAFR-suppressed macrophages. For preliminary tests, we checked whether fluorescent labeling influences infectivity of bacteria which indicated that fluorescent-labeled *B. abortus* hardly affected the efficacy of infection in macrophages (5.64 ± 0.128 CFU (×10^5^)/well in unconjugated bacteria vs 5.12 ± 0.542 CFU (×10^5^)/well in Alexa flour 405-conjugated bacteria). As expected, the stimulation of PAFR by PAF augmented F-actin polymerization upon uptake of *B. abortus* and showed high levels of F-actin rearrangement as much as that of *B. abortus*-infected control cells (Fig. 4, upper and middle panel). However, actin polymerization induced by *B. abortus* uptake was reduced in the CV3988-treated PAFR-suppressed cells (Fig. 4, lower panel). These findings indicated that PAFR contributes to *B. abortus* phagocytosis by boosting F-actin polymerization. Next, we further examined the reorganization of cytosolic activated JAK2 and its colocalization with F-actin during *B. abortus* invasion into PAFR-stimulated or PAFR-suppressed macrophages. The results revealed that the reorganization of activated JAK2 and the colocalization with F-actin were heightened by the stimulation of PAFR and attenuated by the suppression of PAFR (Fig. 4, lower panel). Consequently, these results suggest that PAFR has a positive effect on JAK2 activation-induced actin polymerization for *B. abortus* phagocytosis into macrophages.

**Fig. 5:**
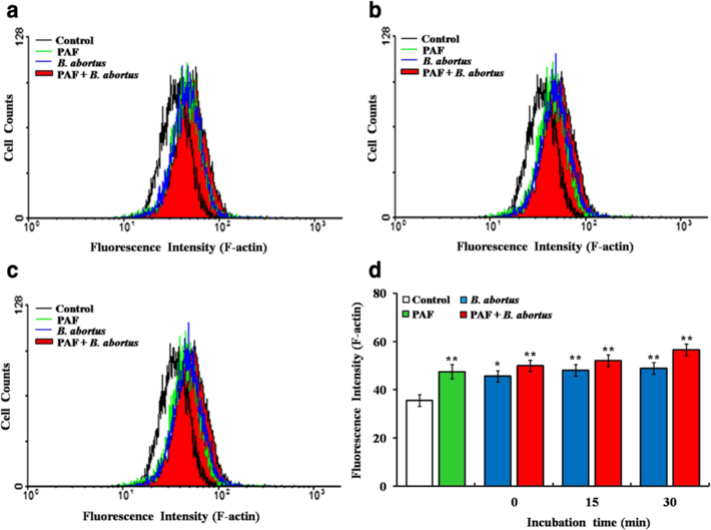
PAFR activation plays a critical role in intensifying F-actin polymerization for phagocytosis of *B. abortus*. RAW 264.7 cells were pretreated for 5 min with PAF (200 nM) followed by infection with *B. abortus* for 5 **(a)**, 15 **(b),** or 30 **(c)** min, and then cells were subjected to FACS analysis for F-actin content. **(d)** The quantitative analysis results of experiment in (**a**-**d**). The average F-actin content of a population was expressed as the mean of the fluorescence intensity. Data represent the mean ± SD of triplicate trials from three independent experiments. Statistically significant differences from the untreated samples are indicated by asterisks (*, *P* < 0.05; **, *P* < 0.01)

Based on the finding of cytoskeletal F-actin redistribution, we evaluated the F-actin content upon *B. abortus* uptake by FACS analysis to quantitatively confirm the action of PAFR on F-actin polymerization for *B. abortus* phagocytosis. The stimulation of PAFR and *B. abortus* infection led to a significant amplification in F-actin fluorescence intensity (*P* < 0.01) and a rightward intensity shift compared with non-stimulated or non-infected cells (Fig. 5). In contrast, the suppression of PAFR resulted in a marked reduction of F-actin content in *B. abortus*-infected cells but not in non-infected cells (Additional file 2: Figure S1). These results suggested that PAFR is associated with the intensification of F-actin polymerization for the phagocytosis of *B. abortus* into macrophages. According to the effects of PAFR activation on *B. abortus* internalization with different infection doses, we additionally investigated F-actin polymerization related to PAFR activation for phagocytosis of *B. abortus* with different infection doses through gating on infected cells. As a result, the intensity of F-actin showed an infection dose-dependent increase and the gating on infected cells have a tendency to increase than that of gating only viable cells (Additional file 3: Figure S2). Collectively, we confirm that PAFR may be a critical signal transduction promoter for F-actin polymerization during *B. abortus* infection in macrophages.

### Trafficking of PAFR from the membrane to endosomes is induced by the uptake of *B. abortus*

PAF-mediated PAFR ligation causes receptor internalization via clathrin-mediated endocytosis (CME) to transduce extracellular signals [23, 24]. Following PAF stimulation, PAFR becomes rapidly desensitized, internalized, and down-regulated, of which pathway is dependent on proteasome, lysosomal pathways and ubiquitination [25]. We assume that *B. abortus* induces the production of PAF for signal transduction via PAFR and subsequent PAF-stimulated PAFR ligation followed by internalization of PAFR from the membrane. To verify this hypothesis, we assessed the amount of peripheral membrane-engaged receptor in membrane isolates from *B. abortus*-infected cells. The membrane-engaged PAFR showed reduced values of 29.37 % in *B. abortus*-infected cells and 37.15 % in PAF-treated cells at 30 min compared to that at 5 min (Additional file 4: Figure S3). Thus, this result indicates that PAFR is internalized from the membrane upon PAF stimulation, which was produced following *B. abortus* infection after 30 min.

**Fig. 6:**
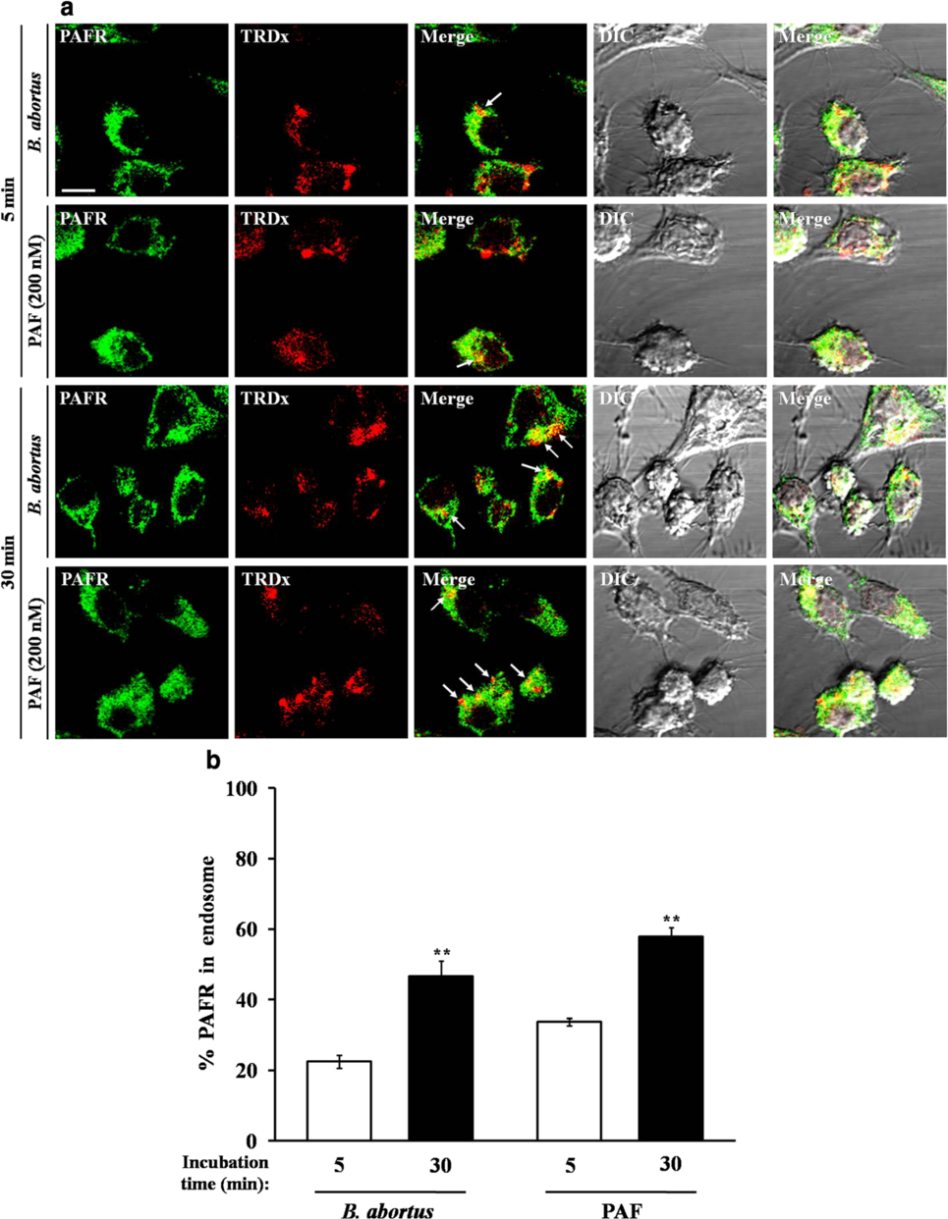
Uptake of *B. abortus* induces trafficking of PAFR to endosomes. **(a)** RAW 264.7 cells were pretreated for 5 min with PAF (200 nM), and then *B. abortus* in medium containing 1 mg/ml of TRDx was deposited onto cells followed by incubation at 37 °C for 30 min. Immunostaining with FITC-labeled PAFR (green) was used to detect the colocalization of PAFR and endosomes (red). The merged images showed colocalization of PAFR and endosomes (arrow). **(b)** The quantitative analysis results of experiment in (**a**). The colocalization number of endosomes with PAFR was counted, and the data are presented as a percentage per 100 cells. All results are representatives of three separate experiments (** *P* < 0.01). All scale bars in images represent 10 μm

Furthermore, we visualized the internalization of PAFR from the membrane and the subsequent trafficking of PAFR to endosomes upon *B. abortus* infection. The levels of colocalization of PAFR with endosomes at 30 min post-infection in *B. abortus*-infected and PAF-treated cells increased to 24.12 % and 24.07 %, respectively, compared to resting cells (Fig. 6a and b). This observation indicates that the increased colocalization of PAFR with endosomes during *B. abortus* uptake is correlated with the reduced amount of membrane-engaged PAFR.

## Discussion

*Brucella* spp. utilize intracellular infection as a virulence strategy and interact with a wide range of host cells, such as macrophages, dendritic cells, and placental trophoblasts [4, 26–28]. The macrophage response to infection causes diverse effects and is critically important for both the endurance of the invasive bacteria and the development of host immunity [2]. Thus, it is important to verify the molecular targets of *B. abortus* in host cells and the virulence strategy used by the bacteria to thwart the host defense responses. In this study, we established an underlying virulence strategy employed by *B. abortus* involving a host cell signaling pathway for phagocytosis of this virulent pathogen. We thus identified the molecular events in the host cell that are essential for the phagocytic strategy used by *B. abortus*.

During the initiation of infection, several macrophage receptors with specific binding sites, including TLR4 [29], that interact directly with *Brucella* have been identified. However, how the receptor enhances phagocytic activity by specific stimuli as a mediator of signal transduction upon infection remains poorly understood.

The invasion of pathogenic organisms into the host elicits a series of immune responses through interactions between a variety of pathogen virulence factors and the immune surveillance mechanisms of the host. Host-pathogen interactions are commonly initiated via host recognition of conserved molecular compositions known as pathogen-associated molecular patterns (PAMPs) [30], which are indispensable for the survival of the pathogen. Detection of PAMPs by a variety of pattern recognition receptors (PRRs) [31], such as Toll-like receptors (TLRs), RIG-I-like receptors (RLRs), and NOD-like receptors (NLRs) triggers the recruitment of diverse adaptor proteins and the activation of downstream signal transduction pathways. This leads to responses essential for defense of the host, including phagocytosis of pathogens, microbial killing, and production of chemokines and cytokines. In addition to the PRRs, another recognition system mediated by PAFR has been reported [9, 32]. PAFR is a GPCR that naturally recognizes the phosphorylcholine determinant on PAF but also recognizes PAMP phosphorylcholine, which results in the uptake of bacteria into host cells. Previous studies concerning the association of PAFR with bacterial phagocytosis have shown that PAFR and its stimulation by ligands exert multiple immunoregulatory reactions by host cells against bacterial infections. These reactions include the promotion of phagocytosis, killing, and cell adhesion [10, 15, 17, 33], suggesting the essential role of PAFR in bacterial invasion. Phagocytosis via PAFR has been shown in several respiratory pathogens that possess phosphorylcholine on their surfaces [34–36]. The trafficking and inflammatory consequences of the interaction of the phosphorylcholine-containing cell wall with PAFR have been determined at the level of cellular physiology as well as in animal models [22]. In contrast, consistent with the fact that *B. abortus* cell wall lacks phosphorylcholine while they have a phosphatidylcholine as a major component of the cell envelope and a requirement for wild-type virulence, we found that colocalization of PAFR with *B. abortus* did not occur during *B. abortus* uptake using confocal microscopy in preliminary test (data not shown). Because a direct contact between PAFR and *B. abortus* was not detected, we thus assessed the effects of a PAFR ligand, PAF, on signal transduction during an entry step in macrophage infection by *B. abortus*.

PAF has diverse immunomodulatory actions for the host defense against bacterial infections, including stimulation and degranulation of granulocytes, monocytes, and macrophages [17, 33]. In an extensive aspect of the relation between PAF and bacteria, we found that *B. abortus* infection led to a remarkable production of PAF during invasion. Furthermore, we postulated that PAF produced upon *B. abortus* infection affects PAFR-mediated events for the advanced uptake of *B. abortus*, hence uptake of *B. abortus* into macrophage was investigated by extrinsic treatment with PAF. The number of internalized *B. abortus* was significantly increased in PAF-stimulated cells, indicating that the stimulated PAFR enhances *B. abortus* uptake into macrophages. This phenotype is dependent on PAF stimulation time as our results show that treatment with PAF for 5 min more significantly increases internalization than treatment for 30 min. In contrast, the inhibition of PAFR function notably reduced the uptake of *B. abortus.* Possibly, the same mechanism occurs in PAFR through *B. abortus*-induced PAF on phagocytosis of *B. abortus* into host cell. Collectively, these findings suggest that the stimulation of PAFR actively influenced *B. abortus* phagocytosis and confirmed that PAFR has the potential to serve as a trigger for the uptake of *B. abortus*.

The PAFR-associated intracellular signaling pathway was found to involve the intracellular tyrosine kinase JAK2, which resulted from the findings that JAK2 fails to become phosphorylated in a PAFR mutant [16, 18]. In agreement with a positive effect of PAFR on JAK2 activation, the phosphorylation of JAK2 was increased by *B. abortus* infection, and the *B. abortus*-induced JAK2 activation was augmented through PAFR stimulation by PAF. In contrast, upon inhibition of PAFR, the elevated JAK2 activation by *B. abortus* infection was obstructed at the internalization stage. Indeed, these findings suggest that PAFR-involved JAK2 activation could be a virulence strategy critical for *B. abortus* invasion into host cells.

It has been known that PAFR activation induces the recruitment of intracellular kinases leading to distinct actin rearrangement at the plasma membrane [22, 23]. To determine if this occurs upon *B. abortus* infection of macrophages engaged in JAK2 activation, we observed the reorganization of the actin cytoskeleton and cytosolic activated JAK2 during *B. abortus* uptake by PAFR-stimulated or -suppressed macrophages. As expected, the microscopy results revealed that the F-actin polymerization and the redistribution of activated JAK2 were augmented by stimulation of PAFR. Furthermore, FACS analysis showed increased F-actin fluorescence intensity upon PAFR stimulation, which correlates with our microscopy results. Therefore, the *B. abortus*-induced effect on actin polymerization enhanced by PAFR-associated JAK2 activation could be essential for macrophage infection by this pathogen. In addition, regarding the infection dose, we verified that the number of internalized bacteria and intensity of F-actin were enhanced in higher infection dose (MOI 100) than in lower one (MOI 10), indicating the effects and levels of PAFR activation on *B. abortus* phagocytosis into macrophage have a tendency to be proportional to the infection dose.

*B. abortus* infection induced intracellular lyso-phosphocholine acetyltransferase (LPCAT) activation, which subsequently participated in PAF synthesis. Advanced studies have shown the complex pathway connecting the stimulation of PAFR through enhanced PAF production by extracellular stimuli which initiates receptor internalization [23, 24]. On the other hand, the experiment to verify whether PAF production linked to LPCAT2 activation is caused by live *Brucella* infection in comparison with killed bacteria and *E. coli* O157:H7 as a non-*Brucella* control revealed that the positive effect on LPCAT2 activation-related PAF production is practically related with live *Brucella* infection but not killed bacteria. But the same positive effects of *E. coli* O157:H7 infection as much as those of live *B. abortus* corresponded with previous study that described a significant increase in PAF production by human colonic tissue for 4 h after infection by enterohemorrhagic *E. coli* (EHEC) [37]. Thus, our results revealed that live *Brucella* infection also induced PAF production through activation of LPCAT2 similar to other bacteria but we particularly verified that this enhancement is linked to the JAK2-mediated pathway. Moreover, the internalization and degradation of PAFR were induced by stimulation of PAF, which can be maintained by proteasome and lysosomal pathways [25]. In line with these signal pathways, the fundamental proposal is that *B. abortus* could induce the production of PAF, which may be advantageous for invasion. Subsequently, the PAFR stimulation by *B. abortus*-induced PAF secretion causes mobilization and trafficking in membrane-engaged PAFR compared with the resting condition. This finding is supported by the evidence showing the increased trafficking of PAFR to endosomes, which is in accordance with the internalization of PAFR from the membrane upon *B. abortus* infection. Considering the relevant evidence that *Brucella* cause a weak response in innate immune system, of which mechanism is likely that *Brucella* do not rely on single distinct virulence factor [38], the event involving PAFR trafficking upon *B. abortus* infection could indirectly regulate PAFR-mediated signaling activation in an integrated aspect of host-pathogen interaction.

Thus, this sequential processing and correlation implies that *B. abortus* utilizes a supporting mechanism (non-binding membrane receptor PAFR), which modulates the activation of PAFR-mediated JAK2 signaling pathway during the entry step of infection.

## Conclusions

In summary, this study concludes that *B. abortus* promotes PAF production, which induces PAFR-linked JAK2 signaling activation to successfully invade macrophages. It seems likely that *B. abortus* may enable us to further elaborate mechanisms of intracellular signaling cascades connected to membrane receptor activation. Thus, we establish a correlation between receptor-mediated cellular signaling and the pathogenic strategy of *B. abortus*.

## Methods

### Cells and culture conditions

The RAW 264.7 murine macrophage cell line was obtained from the American Type Culture Collection (ATCC TIB-71, Rockville, USA) and was grown at 37 °C in a 5 % CO_2_ atmosphere in RPMI 1640 medium (Gibco, Carlsbad, CA) containing 10 % heat-inactivated fetal bovine serum (FBS), 2 mM _L_-glutamine, 100 U/ml penicillin, and 100 μg/ml streptomycin (all provided by Gibco). RAW 264.7 cells were seeded (1 × 10^5^ cells/well) in cell culture plates and incubated for 24 h before infection for all assays.

### Bacterial strains and culture conditions

The *B. abortus* strain used in this study was derived from 544 (ATCC 23448), which is a smooth, virulent *B. abortus* biovar 1 strain. The *B. abortus* organisms were maintained as frozen glycerol stocks (glycerol 80 % v/v) at -70 °C. In all experiments, the contents of freshly thawed vials were cultured in Brucella broth (Becton Dickinson, Franklin Lakes, NJ) or Brucella agar without antibiotics for 3 days at 37 °C with aeration; whereas, enterohemorrhagic *E. coli* (EHEC) O157:H7 (ATCC 43894) cultures were grown in Luria-Bertani (LB) broth. Bacteria were grown at 37 °C with vigorous shaking until they reached stationary phase, and the bacteria were suspended in PBS and then the viable counts were measured by plating serial dilutions on Brucella agar. For generating the killed bacteria, *B. abortus* organisms grown to stationary phase were washed five times in sterile PBS, heat killed at 80 °C for 20 min. Total absence of *B. abortus* viability after heat killing was confirmed by the absence of bacterial growth in Brucella agar.

### Bacterial infection and internalization assay

To detect internalization efficacy of the bacteria, RAW 264.7 cells were infected with *B. abortus* as described previously [5]. In brief, bacteria were inoculated onto cells grown in 96-well plates at multiplicities of infection (MOIs) of 10 and 100, centrifuged at 150 × *g* at 22 °C for 10 min, and were then incubated at 37 °C in 5 % CO_2_ for 0, 15, and 30 min. The cells were washed once with medium and were then incubated with medium containing gentamicin (30 μg/ml) for 30 min to kill the remaining extracellular bacteria. For evaluation of viable bacteria at different time points, the infected cells were washed three times with PBS and were then lysed with distilled water. The number of viable bacteria was determined by CFU counts from serial dilutions of cell lysates on Brucella agar plates. All of the assays were conducted in triplicates and repeated at least three times on different days.

### Immunoprecipitation and immunoblot analysis

*B. abortus*-infected cells in 6-well plates (5 × 10^5^ cells/well) were lysed using ice-cold lysis buffer for 30 min at 4 °C. Total cell lysates were collected by centrifugation at 18,000 × *g* at 4 °C for 15 min. For the isolation of plasma membrane fractions, the infected cells were suspended in 50 mM Tris-HCl buffer (containing 1 mM EDTA, pH 7.4), and then were sonicated on ice 2 times (each for 5 s, at 20 kHz). The sonicated samples were ultracentrifuged at 265,000 × *g* at 4 °C for 2 h, and the resultant pellet (membrane fraction) was resuspended in 50 mM Tris-HCl buffer. After purification of all proteins, protein concentration was determined by Bradford protein assay (Bio-Rad, Richmond, CA). For the immunoprecipitation studies to analyze LPCAT2 activation, the cell lysates were incubated with goat anti-LPCAT2 antibody (1 μg/100 μg of total protein; Santa Cruz Biotechnology, Dallas, TX) overnight at 4 °C. The cell lysates were then mixed with protein G Sepharose beads (Santa Cruz Biotechnology). The final immunoprecipitated product, the initial total cell lysates and plasma membrane proteins were separated by SDS-PAGE and were transferred to a PVDF membrane (Millipore, Billerica, MA). The blots were blocked for 1 h with 5 % (w/v) bovine serum albumin in TBS-T (20 mM Tris-HCl, 150 mM NaCl, Tween 0.1 %, pH 7.4) and were probed by a mouse anti-phosphoserine antibody (1:1000; Millipore) for LPCAT2, rabbit anti-JAK2 (1:2000; Novus, Cambridge, UK) and rabbit anti-p38α (1:1000; Cell Signaling Technology, Danvers, MA), and rabbit anti-PAFR (1:200; Cayman Michigan, USA) phosphospecific antibodies. Pan antibodies and rabbit anti-β-actin antibody (1:1000; Cell Signaling Technology) were used to probe stripped blots to verify that equivalent amounts of proteins were loaded per lane. Mouse anti-Sodium potassium ATPase (Na/K ATPase) antibody (1:1000; Abcam) was applied for quality control of plasma membrane. The binding of primary antibody was visualized using HRP-conjugated anti-rabbit IgG and anti-mouse IgG secondary antibodies (1:5000; Sigma, St. Louis, MO) followed by detection with enhanced ECL (Amersham, Little Chalfont, UK). The immunoblot ECL signals were quantified using NIH Image J software.

### Measurement of PAF content

Cells cultured in 96-well plates were infected with *B. abortus* for 5 min. The PAF concentrations in cell culture supernatants were measured by mouse PAF ELISA (Antibodies-online, Atlanta, GA) according to the manufacturer’s instructions. All assays were conducted in triplicates and repeated at least three times on different days.

### Immunofluorescence microscopy

For using fluorescent-conjugated bacteria, *B. abortus* was labeled with Alexa Fluor 405 (Molecular Probes, Eugene, OR) as follows; Ten mg of bacteria (approximately 10^10^ CFU) was dissolved in 1 ml of 0.1 M sodium bicarbonate buffer (pH 8.3), and then slowly added with 50 μl of Alexa Fluor 405. The reaction mixture was incubated for 1 h at room temperature with continuous stirring. After conjugation, viable bacteria were counted by culture on Brucella agar, it was ascertained that the conjugation of bacteria had no negative effect on viability. Macrophages were cultured in 12-well plates with 18 mm-diameter glass coverslips (10^5^ cells/well) for 24 h before the infection. Cells were infected with unconjugated or Alexa Fluor 405 (Molecular Probes, Eugene, OR)-conjugated *B. abortus* for 5 min. The samples were fixed with 4 % (w/v) paraformaldehyde (Sigma) and were permeabilized with 0.1 % Triton X-100 for 10 min at 22 °C. After 30 min incubation with a blocking buffer (2 % goat serum in PBS), the samples were stained with specific antibodies in blocking buffer for 1 h at 37 °C. For F-actin staining, the cells were incubated with 0.1 μM rhodamin-phalloidin (Cytoskeleton, Denver, CO) for 30 min at 22 °C. For the detection of intracellular JAK2 localization, the cells were incubated with phospho-JAK2 and then FITC-conjugated goat anti-rabbit IgG (Sigma). For detection of colocalization of PAFR and endosomes, *B. abortus* in medium containing 1 mg/ml of TRDx were deposited onto cells by centrifugation at 150 × *g* at 22 °C for 10 min, and were incubated at 37 °C for 5 and 30 min. Immunostaining was conducted with Alexa Fluor 350-labeled PAFR. Finally, the preparations were washed and mounted with fluorescent mounting medium (DakoCytomation, Glostrup, Denmark). Fluorescence images were collected with an Olympus FV1000 laser scanning confocal microscope. The images were processed with Adobe Photoshop and NIH ImageJ software [38].

### FACS assay for F-actin

To determine the relative content of F-actin in cells infected with Alexa Fluor 405-unconjugated or conjugated *B. abortus* (MOIs of 10, 50 and 100), we performed a FACS assay for F-actin as previously described [39]. In brief, the cells (1.5 × 10^6^ cells/ml) were harvested and fixed with 4 % (w/v) paraformaldehyde at room temperature for 30 min. Next, the samples were permeabilized and stained with 20 μg/ml lysophosphatidylcholine (Sigma) containing 1 μM TRITC-phalloidin (Sigma). After centrifugation at 500 × *g* at 4 °C for 5 min, the cells were washed with PBS, and the F-actin content was quantified by FACS analysis using a FACSCalibur flow cytometer (Becton Dickinson, Mountain View, CA). For gating in FACS, two kinds of gating strategy were used; first, the viable cells were gated based on forward-scatter (FSC) and side-scatter (SSC) and F-actin fluorescence was measured by the second plot gated on the viable cells. Second, the population of *B. abortus*-infected cells was drawn to tightly encompass the majority of the viable cells based on FSC and SSC, and F-actin fluorescence was determined by the third plot gated on the infected cells, which was drawn to encompass an expanded area of positive cells. The data were collected as log-scaled fluorescence histograms from 10,000 cells, and the average F-actin content of a population was expressed as the mean of the fluorescence intensity. Experiments were performed in duplicates and repeated at least three times.

### Statistical analysis

The data are expressed as the mean ± standard deviation (SD) for the replicate experiments. Statistical analysis was carried out using Graphpad-Prism software, version 4.00 (Graphpad Software, Inc., San Diego, CA). The Student’s *t*-test or one-way ANOVA followed by the Newman-Keuls test were used to make a statistical comparison between the groups. The results with *P* < 0.05 were considered significantly different. *, **, *** denote *P* < 0.05, *P* < 0.01*, P* < 0.001, respectively.

### Ethics approval and consent to participate

Not applicable.

### Consent for publication

Not applicable.

### Availability of data and materials

The dataset supporting the conclusions of this article is included within the article and its additional files.

## Additional files

Additional file 1:
**Table S1a.** Effect of PAFR activation on *B. abortus* internalization into macrophages. **Table S1b.** Effect of PAFR activation with different infection doses of *B. abortus*. (DOCX 22 kb) Additional file 2: Figure S1. Role of PAFR activation on the intensification of F-actin polymerization for phagocytosis of *B. abortus*. RAW 264.7 cells were pretreated for 1 h with a CV3988 (1 μM), followed by infection with *B. abortus* for 5 **(a)**, 15 **(b)**, or 30 **(c)** min, and then cells were subjected to the FACS analysis for F-actin content. **(d)** The quantitative analysis results of experiment in (a-c). Data represent the mean ± SD of triplicate trials from three independent experiments. Statistically significant differences from the untreated samples are indicated by asterisks (*, *P* <0.05, **, *P* < 0.01). (TIF 1098 kb) Additional file 3: Figure S2. PAFR activation-related F-actin polymerization for phagocytosis of *B. abortus* with different infection doses. RAW 264.7 cells were pretreated with or without PAF (200 nM) for 5 min, followed by infection with *B. abortus* (MOI 10, 50 and 100) for 5 **(a)** and 30 **(b)** min, and then cells were subjected to the FACS analysis for F-actin content. **(c)** The quantitative analysis results of experiment in (a-b). Data represent the mean ± SD of triplicate trials from three independent experiments. Statistically significant differences from the infected cells with *B. abortus* (MOI 10) are indicated by asterisks (*, *P* <0.05, **, *P* < 0.01, ***, *P* < 0.001). (TIF 895 kb) Additional file 4: Figure S3. Uptake of *B. abortus* facilitates the internalization of PAFR from membrane. **(a)** RAW 264.7 cells were pretreated for 5 min with a PAF (200 nM), followed by infection with *B. abortus* for indicated times. Cell plasma membranes were isolated and monitored for the immunoblot analysis using an antibody against PAFR. Sodium potassium ATPase (Na/K ATPase) antibody was applied for quality control of plasma membrane. Images shown are representative of three independent experiments. **(b)** PAFR levels were quantified by fold activation detected as standardized ratio of PAFR to β-actin over basal levels present in resting cells. Data represent the mean ± SD of triplicate trials from three independent experiments. Statistically significant differences from the untreated samples are indicated by asterisks (**, *P* <0.01). (TIF 144 kb)

## Abbreviations

PAFR: platelet-activating factor receptor
JAK2: Janus kinase 2
GPCR: G protein-coupled receptor superfamily
LPCAT2: lyso-phosphatidylcholine acyltransferase 2
MOI: multiplicities of infection
CME: clathrin-mediated endocytosis
TLR: Toll-like receptor: PAMPs, pathogen-associated molecular patterns
PRRs: pattern recognition receptors
