# Supplementary material for: Influence of platelet-activating factor receptor (PAFR) on Brucella abortus infection: implications for manipulating the phagocytic strategy of B. abortus
Source: BMC Microbiol. 2016 Apr 21;16:70. doi: 10.1186/s12866-016-0685-8 (PMC4839150; doi:10.1186/s12866-016-0685-8)
Supplement: Additional file 1: — Table S1a. Effect of PAFR activation on B. abortus internalization into macrophages. Table S1b. Effect of PAFR activation with different infection doses of B. abortus. (DOCX 22 kb) [file 12866_2016_685_MOESM1_ESM.docx]

**Additional file 1**

**Table S1a.** Effect of PAFR activation on *B. abortus* internalization into macrophages.

| Treatment |  | Mean CFU (×10^5^)/well (± SD)^a^ | | |
| --- | --- | --- | --- | --- |
|  | Postinfection time (min) | 0 | 15 | 30 |
| PAF 0 nM |  | 10.00 ± 0.38 | 11.50 ± 0.63 | 12.93 ± 0.54 |
| PAF 50 nM |  | 16.64 ± 0.92^*^ | 16.63 ± 0.76 | 16.07 ± 0.75 |
| PAF 100 nM |  | 16.01 ± 0.99^*^ | 16.50 ± 1.23 | 17.07 ± 0.59 |
| PAF 200 nM |  | 19.79 ± 1.20^**^ | 21.29 ± 1.54^**^ | 21.92 ± 0.59^**^ |
| CV3988 0 µM |  | 10.01 ± 0.40 | 11.49 ± 0.43 | 12.92 ± 0.57 |
| CV3988 0.5 µM |  | 9.78 ± 0.64 | 11.07 ± 0.65 | 12.86 ± 0.93 |
| CV3988 1 µM |  | 7.21 ± 0.43^*^ | 7.57 ± 0.80^*^ | 8.36 ± 0.96^*^ |
| CV3988 2 µM |  | 6.14 ± 0.45^**^ | 6.86 ± 0.88^*^ | 8.86 ± 1.29^*^ |
| Control |  | 10.00 ± 0.48 | 11.54 ± 0.29 | 12.26 ± 0.34 |
| AG490 75 µM |  | 4.99 ± 0.21^***^ | 5.49 ± 0.21^***^ | 6.89 ± 0.38^***^ |
| PAF 200 nM |  | 12.97 ± 0.34^**^ | 14.03 ± 0.15^**^ | 14.85 ± 0.71^*^ |
| AG490+PAF |  | 4.59 ± 0.17^***^ | 5.01 ± 0.23^***^ | 7.28 ± 0.63^***^ |

^a^ Infection dose, 2 × 10^6^ CFU, given to each of three wells per treatment.

All data represent the mean ± SD of triplicate trials from three independent experiments. Statistically significant differences from the untreated samples are indicated by asterisks (*, P < 0.05; **, P < 0.01; ***, P < 0.001)

**Table S1b.** Effect of PAFR activation with different infection doses of *B. abortus*.

| Treatment^b^ |  | Mean CFU (×10^5^)/well (± SD)^a^ | | | | |
| --- | --- | --- | --- | --- | --- | --- |
|  | Postinfection time(min) | 0 | |  | 30 | |
|  | MOI^c^ | 10 | 100 |  | 10 | 100 |
| Control |  | 9.89 ± 0.59 | 31.26 ± 1.61 |  | 12.21 ± 0.42 | 38.07 ± 1.86 |
| PAF |  | 17.68 ± 0.78^**^ | 43.45 ± 1.59^**^ |  | 20.05 ± 0.65^**^ | 47.31 ± 1.46^**^ |
| CV3988 |  | 7.59 ± 0.28^*^ | 20.67 ± 1.33^*^ |  | 8.63 ± 0.55^*^ | 23.53 ± 1.76^*^ |
| AG490 |  | 5.01 ± 0.34^***^ | 17.31 ± 0.95^***^ |  | 7.14 ± 0.44^***^ | 19.58 ± 1.35^***^ |
| AG490+PAF |  | 4.99 ± 0.34^***^ | 20.08 ± 1.07^***^ |  | 7.31 ± 0.69^***^ | 23.45 ± 1.27^***^ |

^a^ Infection dose, 2 × 10^7^ CFU, given to each of three wells per treatment.

^b^Treatment, PAF 200nM; CV3988 1 µM; AG490 75 µM

^c^ Multiplicity of infection

All data represent the mean ± SD of triplicate trials from three independent experiments. Statistically significant differences from the untreated samples are indicated by asterisks (*, P < 0.05; **, P < 0.01; ***, P < 0.001)
