# Supplementary figures and images for: Influence of platelet-activating factor receptor (PAFR) on Brucella abortus infection: implications for manipulating the phagocytic strategy of B. abortus
Source: BMC Microbiol. 2016 Apr 21;16:70. doi: 10.1186/s12866-016-0685-8 (PMC4839150; doi:10.1186/s12866-016-0685-8)

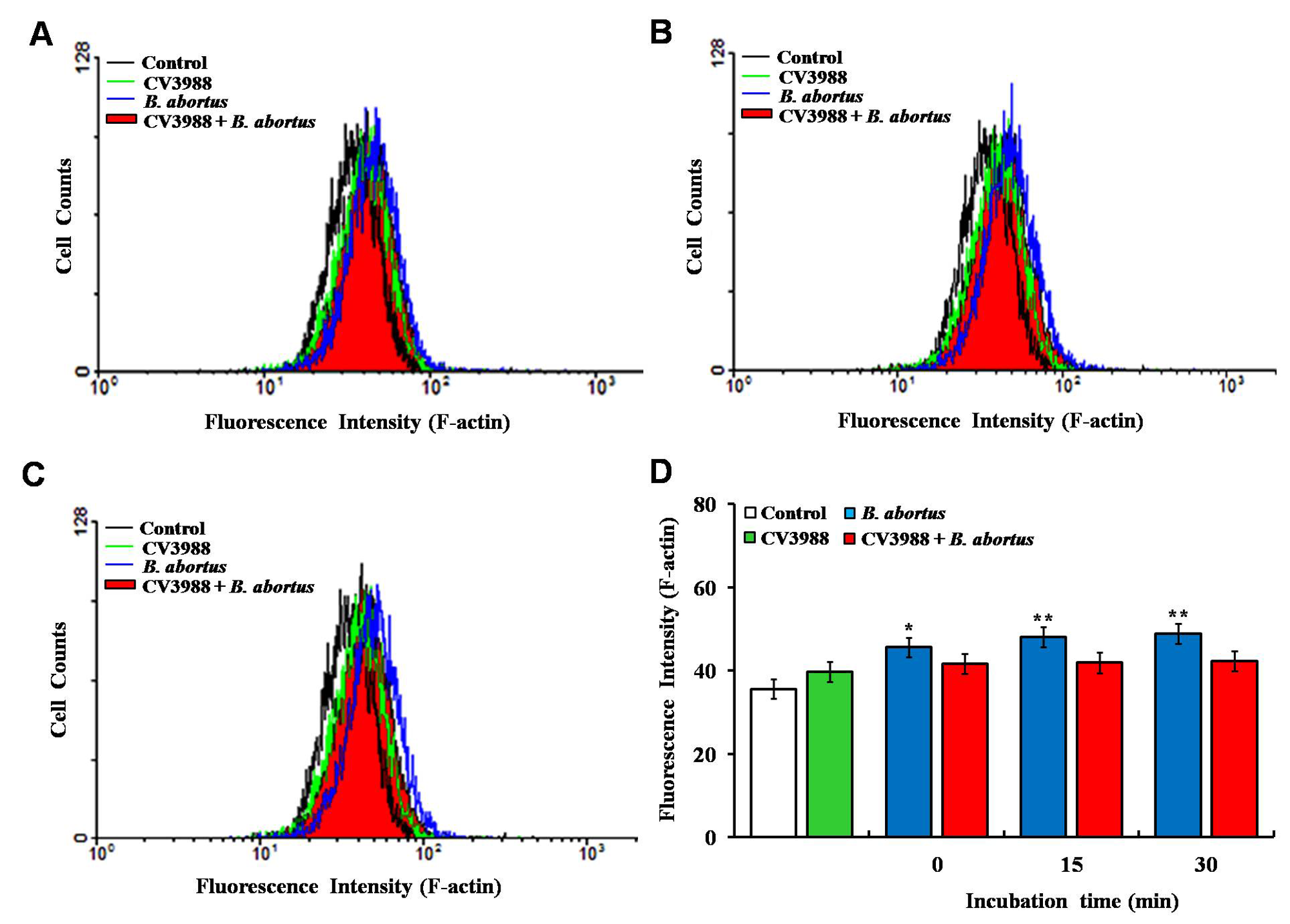

Supplement: Additional file 2: Figure S1. — Role of PAFR activation on the intensification of F-actin polymerization for phagocytosis of B. abortus. RAW 264.7 cells were pretreated for 1 h with a CV3988 (1 μM), followed by infection with B. abortus for 5 (a), 15 (b), or 30 (c) min, and then cells were subjected to the FACS analysis for F-actin content. (d) The quantitative analysis results of experiment in (a-c). Data represent the mean ± SD of triplicate trials from three independent experiments. Statistically significant differences from the untreated samples are indicated by asterisks (*, P <0.05, **, P < 0.01). (TIF 1098 kb) [file 12866_2016_685_MOESM2_ESM.tif]

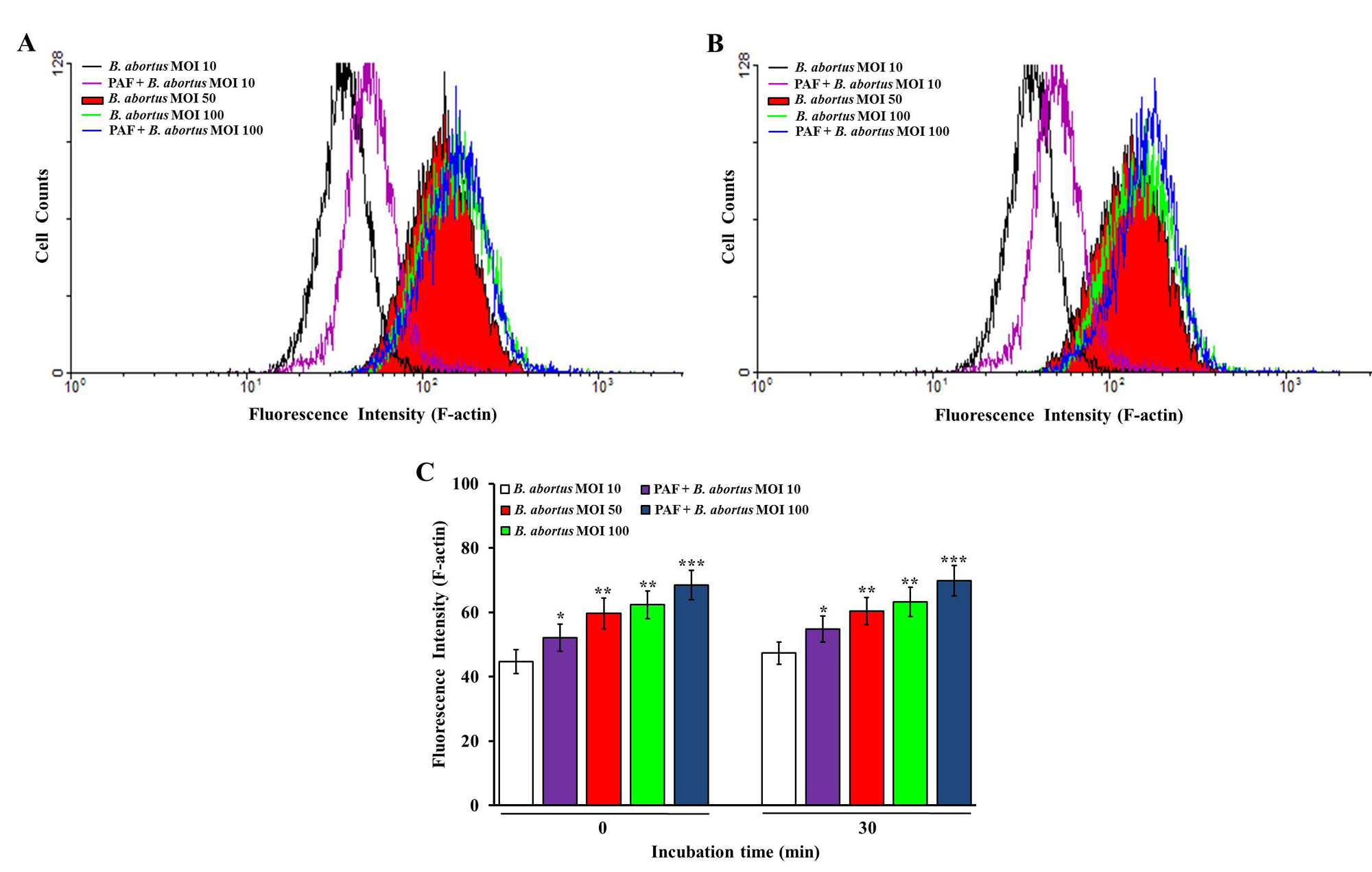

Supplement: Additional file 3: Figure S2. — PAFR activation-related F-actin polymerization for phagocytosis of B. abortus with different infection doses. RAW 264.7 cells were pretreated with or without PAF (200 nM) for 5 min, followed by infection with B. abortus (MOI 10, 50 and 100) for 5 (a) and 30 (b) min, and then cells were subjected to the FACS analysis for F-actin content. (c) The quantitative analysis results of experiment in (a-b). Data represent the mean ± SD of triplicate trials from three independent experiments. Statistically significant differences from the infected cells with B. abortus (MOI 10) are indicated by asterisks (*, P <0.05, **, P < 0.01, ***, P < 0.001). (TIF 895 kb) [file 12866_2016_685_MOESM3_ESM.tif]

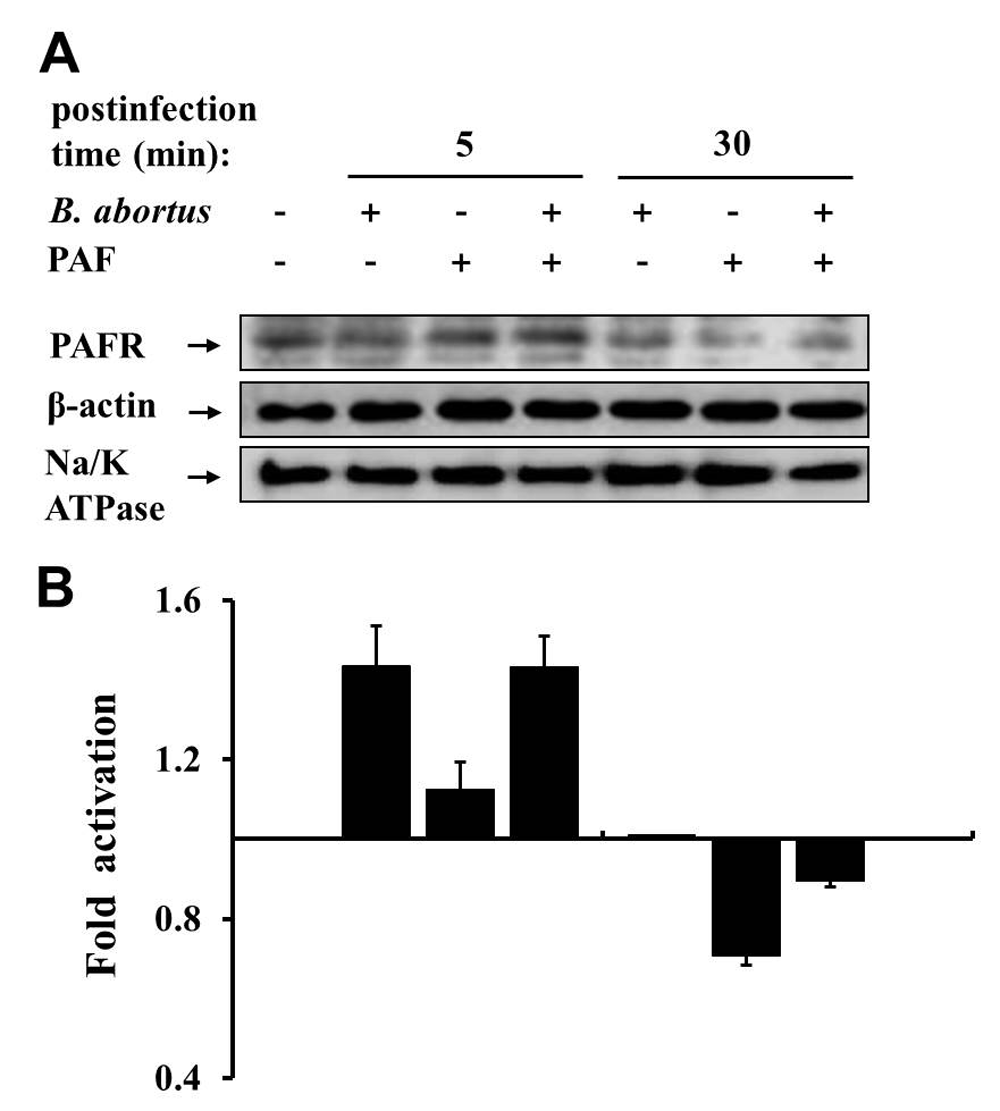

Supplement: Additional file 4: Figure S3. — Uptake of B. abortus facilitates the internalization of PAFR from membrane. (a) RAW 264.7 cells were pretreated for 5 min with a PAF (200 nM), followed by infection with B. abortus for indicated times. Cell plasma membranes were isolated and monitored for the immunoblot analysis using an antibody against PAFR. Sodium potassium ATPase (Na/K ATPase) antibody was applied for quality control of plasma membrane. Images shown are representative of three independent experiments. (b) PAFR levels were quantified by fold activation detected as standardized ratio of PAFR to β-actin over basal levels present in resting cells. Data represent the mean ± SD of triplicate trials from three independent experiments. Statistically significant differences from the untreated samples are indicated by asterisks (**, P <0.01). (TIF 144 kb) [file 12866_2016_685_MOESM4_ESM.tif]
